# PSYCHOMETRIC EVALUATION OF A SWAHILI-TRANSLATED KESSLER 6 AMONG OLDER KENYANS

**DOI:** 10.1093/geroni/igad104.0408

**Published:** 2023-12-21

**Authors:** James Muruthi, Lucy Maina, Elijah Mwega, Violet Kagai, Ndungu Muruthi, Candidus Nwakasi

**Affiliations:** College of Nursing and Health Professions, Drexel University, Philadelphia, Pennsylvania, United States; Kenyatta University, Nairobi, Nairobi Area, Kenya; KARIKA CBO, Nairobi, Nairobi Area, Kenya; Karika CBO, Nairobi, North-Eastern, Kenya; KARIKA CBO, Nairobi, Nairobi Area, Kenya; University of Connecticut, Storrs, Connecticut, United States

## Abstract

The six-item Kessler Psychological Distress Scale (K6) has successfully been used to screen psychological distress, albeit among primarily Western dwelling adults. A major advantage of the K6 scale is the capacity to measure a variety of mental health disorders, such as depression and anxiety. However, limited studies have investigated the applicability of the K6 scale to assess psychological distress among aging individuals in Africa. The purpose of the present study is to evaluate the psychometric properties of a culturally adapted (Swahili version) of the K6 in a sample of aging Kenyans. A sample of 376 participants (N = 376; mean age =69.56 years; 73.7% women) was obtained from three regions in Kenya. Factor analyses were used to examine the properties of the translated K6 in aging Kenyans. The correlation between the scale and other health measures were also estimated using structural equation modeling. Factor analyses revealed a single factor model. CFA demonstrated excellent fit (CFI = 0.950; RMSEA = 0.109). Internal consistency was good (alpha =.83) and the scale had acceptable correlations with chronic (r = .15) and physical health (r = .16). Results underlined that the K6 is a one-factor scale that can be used to measure psychological distress in aging Kenyans. These are promising results for health professionals who seek to conduct population studies on psychological health using a brief scale. Future studies should refine these results to maximize the utility of the K6 scale among aging Kenyans.

